# Sanatorium Troubles

**Published:** 1917-02-03

**Authors:** 


### SANATORIUM TROUBLES.

Cases of complaint against sanatorium management have lately occurred. According to the *Birmingham Daily Post's* report, the secretary of an approved society stated at a meeting of the Birmingham Insurance Committee that in 1916 some 230 patients recommended for sanatorium treatment did not obtain it, because half of them refused to go, and half left prematurely of their own accord. He quoted a specific instance from the signed statement of a patient who stayed three weeks at an institution. It was alleged that there was no medical man in residence, one visiting about every other day, that medicine prescribed was not administered, that the food was bad, and the breakfasts of some patients spoilt by the beds being made during that meal. Complainers lost certain privileges. Continuing, the speaker said that the matron had denied that there were complaints about the food, but admitted that on a few occasions in hot weather the fish had not been quite fresh. He concluded by urging the constitution of a special committee to consider patients' complaints. This advice the Birmingham Insurance Committee is to consider at its forthcoming meeting. In a newspaper interview the next day, more detailed charges were made—dismissal for offences disproportionate or unsubstantiated, such as smoking, card-playing, "alleged gambling"; and that reports of investigations of complaints by the sanatorium sub-committee were only communicated to the general committee member bringing forward the complaint, not to all general committee members.

The lady chairman of the sanatorium sub-committee, speaking in answer to the above, went on very general lines. Sanatorium treatment was tedious and restrictive, patients having much time on their hands; it was very unlike hospital treatment. Complaints might be somewhat numerous in the course of a year, but came from only a small proportion of the total number of patients. She suggested more entertainments to amuse them. The topic then came in for notice in the Press correspondence columns. The medical superintendent of one of the Birmingham sanatoria wrote exonerating his own institution, explaining the need of discipline, and opining that the best sanatorium results ever obtained were those at Nordrach, in Germany. He palliated dismissal for smoking because of the harm it does in tuberculous laryngitis. Mr. Stanton, the society secretary already quoted, replied, expressing a wish to see the Nordrach routine in force at all sanatoria, but showing that the dismissal for smoking he had cited was not on medical grounds at all.

On the whole, it was an instructive discussion, if revealing nowhere great knowledge of sanatorium administration. Mr. Stanton might naturally be a little biased in favour of patients, but the figures he quoted, which were not disputed, certainly furnished him with a *prima-facie* case. His medical interlocutors would as naturally be a trifle pro-doctor. The lady chairman's arguments would have been good enough had not the Birmingham tuberculous scheme been running a good few years now, during the whole of which time the factors she mentioned must have been operative, whilst the friction is apparently of recent date. For ourselves, we find the chief cause of trouble in the absence of a resident physician. Male sanatorium patients, of the working class at least, do not like having to obey a woman. At the time of writing a similar instance has been reported to us, where in the space of a fortnight five men at loggerheads with the matron of a National Insurance sanatorium left one after the other. Complaints from patients, as experienced tuberculosis workers know, in excessive quantity are often merely the sign of a want of proper tone. When about food they mostly come from the most poverty-stricken subjects, or those who are gaining weight at the rate of three or four pounds a week. There is only one way to deal with them. In the Nordrach routine, which was justly praised, the doctor has meals with, and of the same food as, the patients. If then anything is really wrong with what is provided, he sees to it at once; if the complaint be frivolous, he will answer the grumbler with considerable force of manner. At Nordrach there were neither entertainments nor a regular nursing staff; but without the resident physician the routine would have come to a standstill in two or three hours. His hard discipline was manifest to all as rational and altruistic. And that is the whole secret. When adult artisans have to be controlled, as for their health is necessary, in their movements more strictly than any schoolboy, they will not submit to anyone without full medical knowledge, and one, too, who imposes respect upon them by his clinical competence and personal energy; for these are democratic days.

In Nottingham and in the North Riding of Yorkshire has appeared some criticism of sanatorium results as transitory, due to admission of patients too far gone in the disease. As to the particular application of this criticism we say nothing, but in general it is much needed. Unpractised diagnosticians always make the mistake referred to; and probably every tuberculosis officer finds himself every year giving fewer and fewer second-stage cases the benefit of the doubt when selecting for the sanatorium. To admit such is no kindness, even to the sick man himself. Power of early diagnosis, which only comes of application and special study and experience, is therefore the first requisite for a tuberculosis officer. But in how many appointments to such posts is it adequately considered?

